# Immediate Early Genes, Memory and Psychiatric Disorders: Focus on c-Fos, Egr1 and Arc

**DOI:** 10.3389/fnbeh.2018.00079

**Published:** 2018-04-25

**Authors:** Francisco T. Gallo, Cynthia Katche, Juan F. Morici, Jorge H. Medina, Noelia V. Weisstaub

**Affiliations:** ^1^Instituto de Fisiología y Biofísica Bernardo Houssay, Departamento de Fisiología, Facultad de Medicina, Universidad de Buenos Aires (UBA), Buenos Aires, Argentina; ^2^Instituto de Biología Celular y Neurociencias (IBCN) Dr. Eduardo de Robertis, Facultad de Medicina, CONICET, Universidad de Buenos Aires (UBA), Buenos Aires, Argentina; ^3^Departamento de Fisiología, Facultad de Medicina, Universidad de Buenos (UBA), Buenos Aires, Argentina

**Keywords:** Egr1, c-Fos, Arc, fear memory, episodic memory, rodents, psychiatric disorders

## Abstract

Many psychiatric disorders, despite their specific characteristics, share deficits in the cognitive domain including executive functions, emotional control and memory. However, memory deficits have been in many cases undervalued compared with other characteristics. The expression of Immediate Early Genes (IEGs) such as, *c-fos*, *Egr1* and *arc* are selectively and promptly upregulated in learning and memory among neuronal subpopulations in regions associated with these processes. Changes in expression in these genes have been observed in recognition, working and fear related memories across the brain. Despite the enormous amount of data supporting changes in their expression during learning and memory and the importance of those cognitive processes in psychiatric conditions, there are very few studies analyzing the direct implication of the IEGs in mental illnesses. In this review, we discuss the role of some of the most relevant IEGs in relation with memory processes affected in psychiatric conditions.

## Introduction

The diagnoses of psychiatric disorders are based on a cluster of specific symptoms. The genetic, clinical and neuroimaging evidence suggests that they share important features including common affected functions (Bearden and Freimer, 2006). Dysregulation of some mnemonic processes and the mechanisms of neuroplasticity could contribute to prevalent neuropsychiatric diseases. Many psychiatric disorders, despite their specific characteristics, share deficits in the cognitive domain including executive functions, emotional control and memory (Pittenger, 2013). Even though memory deficits have been in many cases undervalued compared with other characteristics, they can be considered as an endophenotype across many psychiatric disorders (Kéri and Janka, 2004; Gur et al., 2007; Henry et al., 2012). Of the many types, episodic and fear memories are two main categories commonly affected in different psychiatric disorders (Dickerson and Eichenbaum, 2010). Episodic memory deficits have been reported in schizophrenia, autism, bipolar disorder, obsessive-compulsive disorder, panic disorder and major depression (Muller and Roberts, 2005; Exner et al., 2009; Brezis, 2015; Czepielewski et al., 2015; Herold et al., 2015; Oertel-Knöchel et al., 2015; Ragland et al., 2015; Vrabie et al., 2015; Meconi et al., 2016; Solomon et al., 2016; Whitton et al., 2016; Green et al., 2017). Fear memories can be linked to anxiety and particularly to post-traumatic stress disorder (PTSD; Parsons and Ressler, 2013; Briscione et al., 2014).

For decades, Immediate Early Genes (IEGs) were used as an indirect marker to measure neuronal activity and, although many of them are routinely measured in thousands of labs around the world, their role in many biological processes is still unknown. The IEGs most commonly used to map neuronal activity, *c-fos* and *Egr1*, have had their expression time-course systematically studied (for mRNA and protein expression dynamics; Mello and Ribeiro, 1998; Bisler et al., 2002; Zangenehpour and Chaudhuri, 2002). They encode transcription factors that influence neuronal physiology by regulating the expression of downstream target genes, normally referred to as late-response genes (Curran and Morgan, 1987; Curran and Franza, 1988; Herdegen and Leah, 1998; O’Donovan et al., 1999; Tischmeyer and Grimm, 1999; Pinaud, 2004; Pinaud et al., 2005). Both c-Fos and Egr1 interact with an array of other transcription factors (Herdegen and Leah, 1998; Knapska and Kaczmarek, 2004). Besides, other IEGs encode proteins that directly influence cellular function, as in the case of *arc* (reviewed in Lanahan and Worley, 1998).

The expression of, *c-fos*, *Egr1* and *arc* are related in literature to learning and memory. Changes in their expression observed in a limited number of cells in regions associated with learning and memory lead to widespread use as readout of plastic changes subserving long-term memory formation and maintenance in specific neuronal populations (Rosen et al., 1998; Guzowski et al., 1999; Ramírez-Amaya et al., 2005; Minatohara et al., 2016). In this review, we focus on the role of, c-Fos, Egr1 and Arc since they have been associated to different memory processes, becoming good candidates as markers or effectors of these processes usually affected in psychiatric conditions.

## Memory and Psychiatric Disorders

Memory is a complex process through which the information acquired during learning is stored. Memory processes can be studied by their stages (acquisition, consolidation, and retrieval), duration (short-term and long-term) and to the type of information that it is stored (explicit and implicit), which are reviewed somewhere else (Eysenck, 1988; Abel and Lattal, 2001; Kandel et al., 2014; Squire and Dede, 2015). We now have significant knowledge and consensus regarding the phases of the memory process as well as some of the key structures, signaling pathways and genes involved in many different types of memories. Though it is still a matter of debate “where” and “how” memories are stored, memories are “stored” as spatiotemporal *representations* within a given neuronal network (Davis et al., 2006). Then, a particular memory might produce an identifiable pattern due to expression of a specific set of activity dependent genes.

Memory consolidation is a process that is proposed to occur at the same synapses involved in the encoding of the information (Dudai, 2002). It includes the activation of transcription factors, protein synthesis, and post-translational modification that lead to plastic changes that make the memory trace stable (Lamprecht and LeDoux, 2004; Morris, 2006; Alberini, 2009; Ruediger et al., 2011). During retrieval, memory reactivation can trigger two quite different processes, *extinction* or *reconsolidation*. The reactivated memory trace becomes labile and is susceptible to being modified or disrupted in the process called reconsolidation. If there is a reinforcement of the original trace during retrieval, the reconsolidation—a protein synthesis-dependent process—re-stabilizes the original memory trace (Lee, 2008). On the contrary, after repeated reactivations in the absence of the appropriate reinforcement, memory extinction is triggered, leading to the loss in memory expression. This process is also protein-synthesis dependent and evidence shows that a new memory trace is formed, replacing the original one without erasing it (Quirk and Mueller, 2008). Then, it emerges that from the clinical point of view, it is important to understand the underlying mechanisms of these phenomena in order to develop new tools as novel treatment in Psychiatry (Monfils et al., 2009; Schiller et al., 2010).

In psychiatric disorders, episodic, working and fear memories are the most commonly affected types of memories, making them relevant to be studied in animal models (Dickerson and Eichenbaum, 2010; Parsons and Ressler, 2013; Briscione et al., 2014; Oertel-Knöchel et al., 2015; Meconi et al., 2016; Whitton et al., 2016; Martinussen et al., 2005; Rasetti and Weinberger, 2011). Episodic memories encode series of events that occur at a particular place and a specific time. Despite that episodic memories bear a strong anthropocentric character, it is now accepted that some characteristics of episodic memory, like recognition, are found in different species, allowing the study of their mechanisms and substrates in different models (Binder et al., 2015; Morici et al., 2015).

Working memory is the ability to maintain current representations of goal relevant knowledge. It is an executive component that is distributed across the frontal lobe together with sensory cortices of various modalities which interact through attentional processes (Postle, 2006; Carruthers, 2013). Over the years, working memory tasks were developed to allowed the study of working memory, its processes and neural substrates in different animal models (Carruthers, 2013; Dudchenko et al., 2013).

Although the expression of fear in animals might not have its exact correlate in humans, fear memories have been extensively studied in animal models to identify the neurobiological basis underlying memory processes (LeDoux, 2014; Izquierdo et al., 2016). Historically, the most common tasks used to study fear memories have been fear conditioning and inhibitory avoidance tasks. Both behavioral approaches are associative learning tasks, were an “unconditioned stimulus” (context or cue) is linked to an aversive “conditioned stimulus” (shock) resulting in a “conditioned response” (freezing or place avoidance). These kinds of tasks allowed the study of the molecular mechanisms and brain structures involved in the processing of fear memories.

## Immediate Early Genes in Learning and Memory

IEGs represent a class of genes that respond rapidly and transiently to a variety of cellular stimuli (Hughes and Dragunow, 1995; Kaczmarek and Chaudhuri, 1997; Herdegen and Leah, 1998; Pinaud et al., 2005; Terleph and Tremere, 2006; Bahrami and Drabløs, 2016). There are more than 100 genes classified as IEGs (Sheng and Greenberg, 1990; Minatohara et al., 2016), although only a small subgroup was found in neurons (Sheng et al., 1995). Since the discovery of the regulation of IEGs in the brain by neural activity, there has been an extensive research using IEGs as neural activity markers in studies of behavior and cognition. However, there is some controversy regarding the physiological role that these genes might have. Over the years, different hypotheses were put forward. For example, some groups assigned IEGs a role in homeostatic maintenance or replenishment, while others found in IEG’s expression a role for the maintenance of activity dependent plastic changes (for review Dragunow, 1996). Another hypothesis suggested that they might be involved in mechanisms of information integration (Kaczmarek, 2000). Independently of the role that these genes have at a cellular level, it is clear that their expression is affected by neuronal activity and they are used as neuronal activity markers. In particular, some studies have reported changes in IEG expression associated with learning and memory processes and in connection with psychiatric disorders. In the next subsections we will discuss three of the most common IEGs studied in relation with memory processes and psychiatric disorders.

## Molecular Mechanisms of c-Fos

c-Fos was one of the first transcription factors whose induction was shown to be activity-dependent (Morgan and Curran, 1988; Sagar et al., 1988). In neurons, *c-fos* expression appears to be stimulated by cAMP and Ca^2+^ through the activation of the CREB/CRE complex. The *c-fos* gene codes for the Fos protein that dimerizes with transcription factors of the Jun family to build up the transcription factor AP-1 (Chiu et al., 1988; Pennypacker, 1995).

## c-Fos and Memory

*c-fos* is a clear example of an IEG whose increased expression is routinely used as an indicator for neuronal activation. For instance, it has been shown that *c-fos* expression following behavioral training specifically correlates with learning (Maleeva et al., 1989, 1990; Kaczmarek and Nikołajew, 1990; Tischmeyer et al., 1990), performance (Sakai and Yamamoto, 1997; Radulovic et al., 1998; Bertaina-Anglade et al., 2000; Vann et al., 2000a,b) and with cellular ensembles that are activated following memory retrieval (Maviel et al., 2004; Kubik et al., 2007; Lopez et al., 2012; Bravo-Rivera et al., 2015), supporting its role as activity marker.

Interestingly, changes in c-Fos expression were observed mainly during the first sessions of multiple-session training protocols, indicating an adaptive response (Maleeva et al., 1989, 1990; Kaczmarek and Nikołajew, 1990; Nikolaev et al., 1992a,b; Hess et al., 1995a,b, 1997; Gall et al., 1998; Lukasiuk et al., 1999; Anokhin et al., 2000; Bertaina-Anglade et al., 2000).

Genetic manipulations of the *c-fos* gene were performed during the first genetic engineering Era. One of the first approaches aimed to analyze the function of c-Fos in memory was done using whole-body knockout mice. With this model they were found deficits in complex but not in simple behavioral tasks, indicating that *c-fos* is not necessary for all types of learning tasks and memories. It is important to point out that these animals showed many developmental malformations which might affect the interpretation of the behavioral studies (Paylor et al., 1994).

Using the cre/loxP system Fleischmann et al. (2003) developed a central-nervous-system-selective knock out mouse. This model had normal locomotion and emotional related responses but was impaired in hippocampus-dependent spatial and associative learning tasks, such as Morris water maze and contextual fear memory. The mutant mice displayed a reduction of synaptic plasticity mechanisms in hippocampal CA3-CA1 synapses. These specific deficit were consistent with cued and spatial tasks in which an increase in hippocampal c-Fos expression was observed (Guzowski et al., 2001a) and also with experiments done with *c-fos* antisense oligonucleotide (ASO) in rats in which the infusion of the ASO produce deficits in spatial long-term memory (Guzowski, 2002; Kemp et al., 2013).

Fear memory paradigms were also able to produce changes in c-Fos expression in different structures: using an inhibitory avoidance task (Bekinschtein et al., 2010; Katche et al., 2010; Katche and Medina, 2017). It was shown that fear memory persistence required c-Fos expression and that blockade of c-Fos expression by infusion of *c-fos* ASO into the dorsal CA1 region of the hippocampus or retrosplenial cortex produces deficits in the consolidation and persistence of this type of memory (Katche et al., 2010, 2013; Katche and Medina, 2017). Similarly, the infusion of c*-fos* ASO in the prefrontal cortex affected long-term cued fear memory (Morris and Frey, 1999).

The results described here concerning c-Fos expression and function in learning and memory still do not answer the question of the biological role of c-Fos within the brain. Then, is c-Fos expression a response to the activity of neurons within a memory circuit? Or, does it play a role in maintenance of cellular homeostasis? An important obstacle in solving these questions is that the role of c-Fos in synaptic plasticity is still unclear. c-Fos is part of a complex, the AP-1, whose target genes downstream have yet to be fully characterized. Independently of the mechanism operating after c-Fos activation, it is clear that understanding its role at the cellular level is more complex than for other IEGs, such as *narp*, *homer1a* and *arc*, that are known to encode proteins that have a direct effect on the synapses (Chowdhury et al., 2006; Chang et al., 2010; Roloff et al., 2010; Lu et al., 2013). Still, it is important to increase our knowledge of the role of c-Fos at the cellular level to be able to address if it is really a marker of specific neuronal engrams associated with specific memory episodes.

More recently, the *c-fos* promoter was combined with optical sensitive proteins to mark and manipulate a particular subset of cells involved in contextual learning. With this technique, Cowansage et al. (2014) found that c-Fos-expressing neurons in retrosplenial cortex are involved in the acquisition of contextual memories and that the reactivation of this particular set of cells can control behavioral response. Using a similar approach Liu et al. (2012) attempted to test the existence of memory engrams with a strategy of optogenetic and temporary control of cellular activation. Their results lead to two important conclusions. First, they provide evidence that IEGs, combined with optogenetics, can tag neurons that are activated during memory encoding for later manipulation. Second that artificial light-reactivation of these memory engrams is sufficient for behavioral memory recall for contextual fear conditioning. These results support the role of c-Fos in learning and memory while, at the same time demonstrate the power that this type of approach have to understand memory processing in more detail. If it is combined with other genetic models, it can become an extraordinary tool for better understanding the functional link between IEG, memory (Semon, 1904; Morris, 1999; Martin and Morris, 2002; Gerber et al., 2004; Josselyn, 2010) and psychiatric disorders (Chowdhury et al., 2006; Chang et al., 2010; Roloff et al., 2010; Lu et al., 2013).

## Molecular Mechanisms of Egr1

Egr1, also known as Zif-268, NGFI-A, Krox 24 or ZENK (Milbrandt, 1987; Lemaire et al., 1988), is a member of the zinc finger family of transcription factors. Its expression can be induced by a variety of signals that includes injury, stress, differentiation factors, as well as extracellular signals like peptides, neurotransmitters and growth factors (Herdegen and Leah, 1998; O’Donovan et al., 1999; Davis et al., 2003; Clements and Wainwright, 2010). Compared to *c-fos*, *Egr1* has a distinct pattern of expression in the brain (Milbrandt, 1987; Herdegen et al., 1990; Mack et al., 1990; Waters et al., 1990) and mediates the expression of a number of late-response genes involved in different neuronal processes from growth control to plastic changes (Sukhatme et al., 1988; Williams et al., 2000; Bozon et al., 2003; Maddox et al., 2011). *Egr1* has a relatively high expression maintained by normal ongoing neuronal activity (Worley et al., 1991; Beckmann et al., 1997; Herdegen and Leah, 1998) in the hippocampus (Hughes et al., 1992; Cullinan et al., 1995; Okuno et al., 1995; Desjardins et al., 1997), including the dentate gyrus (Cole et al., 1989; Wisden et al., 1990), as well as other brain regions (Herdegen et al., 1990, 1995; Cullinan et al., 1995; Okuno et al., 1995).

## Egr1 and Memory

The first approach to study Egr1 expression and its relation with behavior employed two-way avoidance training (Nikolaev et al., 1992b). In this study it was shown, for the first time, an increased level of *Egr1* mRNA in the hippocampus after one training session (Nikolaev et al., 1992a). In general, most studies use fear conditioning paradigms. With fear conditioning, *Egr1* is rapidly induced by behavioral training in the amygdala (Rosen et al., 1998; Kwon et al., 2012), the hippocampus (Nikolaev et al., 1992a; Miyashita et al., 1998; Guzowski et al., 2001b) and the retrosplenial cortex (Pothuizen et al., 2009). Nonetheless, inconsistent results were also found: Hall et al. (2000) reported non-specific expression of this IEG. Similarly Weitemier and Ryabinin (2004) showed a lack of *Egr1* expression in the septum, amygdala, hippocampus and the anterior cingulate cortex when studying fear conditioning in C57BL/6J mice.

Despite these discrepancies, it is accepted that Egr1 has an important role in learning and memory. Deletion of *Egr1* produces impairment across a broad number of behavioral tasks related to different brain regions. Typically, *Egr1* mutant mice displayed intact short-term memory in several types of tasks; however, long-term memory was drastically impaired in tasks such as social transmission of food preference, taste aversion memory, spatial memory, object recognition memory and object-place recognition memory (Jones et al., 2001; Bozon et al., 2003; Davis et al., 2010). Interestingly, studies using ASOs to partially knock down Egr1 in specific structures showed that the knockdown of Egr1 in the hippocampus does not impair contextual fear conditioning (Lee et al., 2004) but impairs inhibitory avoidance memory persistence (Katche et al., 2012) as well as recognition memory (Zalcman et al., 2015). Egr1 knockdown in the amygdala impairs both contextual (Malkani et al., 2004) and cued-fear memory formation (Maddox et al., 2011) suggesting that the deficits observed in the knockout might be attributed to its role in specific structures or circuits. *Egr1* knockout mice were also shown to be impaired in the consolidation and reconsolidation of contextual fear memory, while heterozygous mice showed impairment only in the reconsolidation (Besnard et al., 2013). This difference supported the hypothesis that memory reconsolidation is not mechanistically a repetition of consolidation. Consistent with the phenotype observed in knockout mice, overexpression of *Egr1* improved spatial memory, but not memory for the objects, during a recognition memory task (Penke et al., 2014) and enhanced aversive memories’ resistance to extinction (Baumgärtel et al., 2008). On the other side, failure to induce *Egr1* allowed spontaneous recovery of fear memory after extinction (Herry and Mons, 2004).

Historically, a number of studies supported the view that proposed that *Egr1* expression is sensitive to information gained after the exposure to novelty or learning associated environments (Tischmeyer and Grimm, 1999; Bozon et al., 2002; Guzowski, 2002; Davis et al., 2003, 2006; Knapska and Kaczmarek, 2004). This idea came from the increments in the expression of *Egr1* mRNA or protein after different learning paradigms. Some examples are cited above, others are: brightness discrimination (Grimm and Tischmeyer, 1997), visual paired associate learning in monkeys (Okuno and Miyashita, 1996; Tokuyama et al., 2002), birds song learning (Mello et al., 1992; Jarvis et al., 1995; Bolhuis et al., 2000), learning and retrieval of contextual and cue fear memory (Frankland et al., 2004; Weitemier and Ryabinin, 2004) and spatial learning (Guzowski et al., 2001b). Mechanistically, a role of Egr1 in learning and memory is supported partially by its modulation of plastic changes including spine and synapse remodeling as well as growth of new synaptic connections (Lamprecht and LeDoux, 2004; Miniaci et al., 2008; Lai et al., 2012).

These studies support a role of Egr1 in processes of learning and memory formation and they do determine a *necessity* of Egr1 for these neural and cognitive functions. But what is the specific role of Egr1 in plasticity or memory processes? It is a highly regulated transcription factor with many identified target genes and probably many more still unknown. Unlike c-Fos, there is a multitude of genes related to vesicular transport and neurotransmitter release, clathrin-dependent, or actin, which are commonly observed as direct Egr1 targets (Koldamova et al., 2014; Duclot and Kabbaj, 2015, 2017b), supporting its role in synaptic plasticity and, through this mechanism, in learning and memory. Then, alterations in the normal expression of *Egr1*, or in its protein function, could affect the encoding of information in the engram and, therefore, affect higher orders of organization.

## Molecular Mechanisms of Arc

The activity-regulated cytoskeletal (Arc) protein also known as Arg3.1, is one of the most characterized molecules involved in the consolidation of different types of memories. In contrast to *c-fos* or *egr1*, this gene is known to code for a synaptic protein. Arc is one of the effectors of the BDNF, glutamatergic, dopaminergic and serotonin signaling (Chowdhury et al., 2006; Granado et al., 2008; Karabeg et al., 2013; Leal et al., 2014; Panja and Bramham, 2014; Pastuzyn and Keefe, 2014; Managò et al., 2016; Mastwal et al., 2016). *arc* expression is under regulation of Egr1 (Li et al., 2005). It is well characterized that the *arc* mRNA is transported to the dendrites (Fujimoto et al., 2004; Steward et al., 2015) and is usually used as marker for neural activity (Chowdhury et al., 2006; Shepherd et al., 2006; Li et al., 2015; Ivashkina et al., 2016). In this sense, the post-synaptic dendrites are enriched with *arc* mRNA in contrast to the absence at the pre-synaptic axons (Moga et al., 2004; Dynes and Steward, 2012; #6010; Steward et al., 2015). Arc is involved in the generation of new synapses and the maintenance of old ones required for some plasticity mechanisms such as long-term potentiation (LTP) and long-term depression (LTD; Korb and Finkbeiner, 2011; Minatohara et al., 2016). Besides, *arc* encodes a growth factor that associates with F-actin (Lyford et al., 1995). These features led to postulate that Arc is involved in experience-dependent dendritic reconfiguration (Pinaud et al., 2001; Steward and Worley, 2001; Pinaud, 2004).

*Arc* gene has specific sequences normally found in retroviruses such as HIV (Campillos et al., 2006). Recent evidence was found of a plausible novel molecular mechanism by which genetic information could be transferred between neurons. It was shown that Arc protein forms a virus-like particle that can enclose RNA and be transferred through the synapse (Pastuzyn et al., 2018). Interestingly it was also proven in *Drosophila* (Ashley et al., 2018). Though the mechanism is not entirely new, considering there have been studies suggesting these pathways and their potential role in synaptic plasticity before Budnik et al. (2016); Zappulli et al. (2016) this was the first time that Arc was proven to form these particles and enclose RNA. Further research is needed on this mechanism to understand how it can be linked with synaptic plasticity.

## Arc and Memory

Up-regulation of *arc* in the Morris water maze was observed in cortical and para-hippocampal regions during memory spatial retention and after fear conditioning training, triggered by context exploration (Lonergan et al., 2010; Barry et al., 2016). Gusev and Gubin (2010) found differences in *arc* expression with memory durability; select segments in the prefrontal, retrosplenial, somatosensory and motor cortex showed similar robust increases in *arc* expression in recent and remote spatial memories. In another work, the study on the expression of *arc* yielded its requirement for the consolidation of long-term memory, but not for learning or short-term memory (Plath et al., 2006). Kubik et al. (2012) showed that the inactivation of dorsal CA1 was sufficient to impair the spatial performance in the Morris water maze task and this was also followed by a reduction of the expression of *arc* in the retrosplenial cortex. The requirement of Arc expression for long-term plasticity and memory consolidation was shown by infusing *arc* ASO into the dorsal hippocampus, the lateral amygdala or the anterior cingulate cortex (Guzowski et al., 2000; Ploski et al., 2008; Holloway and McIntyre, 2011; Nakayama et al., 2015). Arc also facilitates the consolidation of weak memories, and has been reported to play a role in behavioral tagging in the hippocampus (Moncada and Viola, 2007; Ballarini et al., 2009; Wang et al., 2010; Moncada et al., 2011; Martínez et al., 2012).

A genetic KO for *arc* expression generates deficits in the consolidation of different types of long-term memories (spatial, fear and episodic-like) together with changes in long-term plasticity (Plath et al., 2006; Peebles et al., 2010; Yamada et al., 2011). In addition, a significant correlation between *arc* mRNA expression and behavioral performance was found during spatial reversal task suggesting a role in cognitive flexibility (Guzowski et al., 2001b). The same group, Guzowski et al. (2006) showed that both the activity and the *arc* dynamic expression of CA1 neurons depend on the recent behavioral history of the animal: when the animals were exposed repeatedly to the same context, *arc* expression in the CA1 region was decreased. A recent study showed that the infusion of *arc* ASO into the perirhinal cortex in an object-pattern-separation task affects the consolidation of overlapping object memories (Miranda and Bekinschtein, 2018).

In summary, of the three genes reviewed here, a large and robust body of evidence suggests that *arc* and *Egr1*, although to a different extent, influence the dynamics of large networks associated with learning and memory, indicating that they have a more specific role than *c-fos*.

## Psychiatric Disorders: Memory Deficits and IEGs

Going from genes to neuropsychiatric disorders has proven to be a hard task, partly because psychiatric disorders have polygenic origin with increasing levels of complexity. Each gene will have an effect at a cellular level but it will also interact with other genes with its own regulation.

Another relevant issue is the environmental factor. It is well established that complex interactions between genes and the environment are involved in multiple aspects of neuropsychiatric disorders. It can determine the vulnerability to a particular disorder and even the response to therapeutic intervention. Then, it seems crucial to achieve a better comprehension of the reaction of individuals to environmental stimuli in a particular genetic context. This level of analysis exceeds the scope of this review. Here we will approach how IEGs can intervene with fundamental neurobiological mechanisms of behavior, bearing in mind that in the central nervous system neuronal plasticity and neurotransmission are among the main processes of interactions between genes and the environment. In particular, IEGs are critical components of these interactions for they provide the molecular framework for a rapid and dynamic response to neuronal activity, opening the possibility of a lasting and sustained adaptation by regulating the expression of a wide range of genes.

The defining characteristics of the IEGs, i.e., activation within the range of minutes and independent expression of protein synthesis, give us a very strong guideline that they are a necessary mechanism of action that will, in turn, allow encoding and storing of memories. Then, deciphering the functions of IEG can provide relevant information on how these mechanisms fail in pathological conditions and thus provide new insights into the molecular mechanisms that are responsible for symptoms associated with neuropsychiatric disorders.

## IEGs in Major Depression Disorder

Major depressive disorder (MDD) is a mood disorder with prominent disturbances in cognitive functions such as certain types of memory, executive function, and attention (Jaeger et al., 2006; Mcintyre et al., 2013). MDD patients have reduced hippocampal and prefrontal cortex volumes. These alterations are mainly due to a clear-cut reduction in the neuropil and neurons in both regions have less complex dendritic trees (Saylam et al., 2006; Arnone et al., 2012). Animal models of depression have also shown a reduction in the volume of some regions of the prefrontal cortex and hippocampal formation (Hains et al., 2009; Czeh et al., 2016) with decreases in the length and complexity of dendrites and reduced number of dendritic spines.

One IEG that participates in synaptic transmission and dendritic plasticity is *arc*. Given that it has been shown that the expression of *arc* in the hippocampus and prefrontal cortex in animal models of depression change in a similar way to the alterations found in post-mortem tissues of MDD patients (Lee et al., 2012), it was postulated as a candidate target for intervening to ameliorate cognitive deficits in MDD. Interestingly, chronic stress *reduces* *arc* expression in medial prefrontal cortex and increases it in the amygdala (Ons et al., 2010), mirroring what is observed in MDD patients (Lee et al., 2012). Interestingly, chronic—but not acute—treatments with antidepressants restore *arc* expression in the hippocampus and prefrontal cortex (Pei et al., 2003; Molteni et al., 2008, 2010) supporting its involvement in psychiatric disorders.

Another IEG associated to MDD is *Egr1*. Covington et al. (2010) found a decreased expression of *Egr1* in the medial prefrontal cortex in depressed patients refractory to treatment and it was also observed in non-medicated subject. It should be noticed that this region has consistently been reported to be affected in depressed patients and in animal models of depression (Krishnan and Nestler, 2008; Koenigs and Grafman, 2009; Duclot and Kabbaj, 2017a,b; Lefaucheur et al., 2017). Based on these and other studies, a direct link between *Egr1* in the mPFC and the depressive phenotype had begun to be analyzed as a possible marker to predict the effectiveness of antidepressants (Morinobu et al., 1995, 1997; Bjartmar et al., 2000; Duclot and Kabbaj, 2017a,b).

Repeated exposure to stressful experiences is one of the main risk factors for the development of stress-related mood disorders like anxiety and depression (Kessler and Wang, 2008). Depending on the nature, duration, and intensity of the stress, changes in the expression of *Egr1* may vary across the entire central nervous system (Knapska and Kaczmarek, 2004). In addition, Egr1 is a critical factor in encoding the behavioral enduring effects of stress in the hippocampus. Moreover, stress-related fear memory is associated with an increased expression of Egr1 and the fear related response is blocked by knocking down Egr1 expression (Revest et al., 2005, 2010; Saunderson et al., 2016).

In line with this clinical observations, it was found that chronic stress provoked in rodents a decreased expression of *Egr1* in the medial prefrontal cortex (Covington et al., 2010) and hippocampus (Xu et al., 2015), and an increase of *Egr1* expression in the lateral amygdala (Monsey et al., 2014). In addition, using social isolation in rodents, another animal model of depression, it was observed a marked reduction in *Egr1* mRNA levels in the hypothalamus, the hippocampus, and the medial prefrontal cortex (Matsumoto et al., 2012; Hodges et al., 2014; Okada et al., 2014). This can be paralleled to the general idea that stress-related depressive disorders are associated with a down-regulated activity in the prefrontal cortex and the hippocampus, and an up-regulation of neuronal activity in the amygdala.

A new player in the neurobiology of depression is another member of the FOS family, ΔFosB. In marked contrast to what happens with the other members of the family, this transcriptional regulator is not rapidly activated by environmental stimuli. Instead, its activation is delayed and accumulates to repeated stimulation (chronic stress) mainly in nucleus accumbens neurons (Perrotti et al., 2004; Nestler, 2008). In addition, chronic administration of antidepressants, like fluoxetine and ketamine, induce ΔFosB in nucleus accumbens (Vialou et al., 2010; Donahue et al., 2014), a brain region associated to reward and motivation. Nestlers group has established that increased expression of ΔFosB within accumbal D1-type medium spiny neurons promotes stress resilience, and mediates antidepressant-like responses (Nestler, 2015). In addition, ΔFosB overexpression in nucleus accumbens promotes several rewarding behaviors, including sucrose drinking, consumption of high-fat food, and sexual activity. Despite its role in nucleus accumbens associated behaviors, since depression is a complex disorder affecting many brain regions, it will be important to study ΔFosB role in other brain structures in which its expression is induced by chronic antidepressant administration (Vialou et al., 2014).

## Fear-Related Disorders and the Involvement of IEGs

Most anxiety disorders are associated with a strong environmental component (fear) and a physiological defect in response to that component. Anxiety disorders include simple phobia, social phobia—which involves fear and avoidance of social situations—, and panic disorder; they all share abnormal fear responses associated with different environmental stimuli but also differ in important and specific symptoms. For example, PTSD is a chronic neuropsychiatric disorder that results from a very strong traumatic event and it is characterized by intrusive and persistence fear related memories. The etiology of PTSD remains largely unknown but different neurobiological systems have been identified as participants in the disorder (Parsons and Ressler, 2013).

The first opportunity to apply treatments designed to modify the formation and persistence of fear memories is around the aversive experience, mainly through pharmacological manipulations (Monfils et al., 2009; Quirk and Milad, 2010; Quirk et al., 2010). Modulation of opioid systems has been proven to be effective in PTSD (Holbrook et al., 2010). However, other pharmacological manipulation yielded mixed results (Maren and Chang, 2006; Myers et al., 2006; Parsons and Ressler, 2013). A new strategy was developed to attenuate fear-related memories by manipulating memory reconsolidation and extinction processes. Immediate or delayed extinction procedures induce a reduction in those aversive memories that are context-dependent and short-lived (Hermans et al., 2006; Woods and Bouton, 2008) suggesting that its efficacy as a treatment depends on how old the memory is (Milekic and Alberini, 2002; Suzuki et al., 2004). A novel behavioral design that involves a mixed reconsolidation-extinction procedure has proved to be a better strategy (Monfils et al., 2009; Chan et al., 2010; Schiller et al., 2010).

Few studies have analyzed the role of IEG in PTSD related animal models, though they reported interesting results. Changes in *arc* expression was observed along the septo-temporal axis of the hippocampus of PTSD susceptible rats (Nalloor et al., 2014), suggesting that it might exist a basal difference among susceptible population. *arc* expression in the hippocampus is also involved in the perpetuation of fear related memories (Nakayama et al., 2015) while changes in the amygdala appeared to be related to the response to uncontrollable stress (Machida et al., 2018). Then, the IEGs analyzed here directly or indirectly play a role in these processes, so that a better understanding of the molecular mechanisms underlying memory processing, including the role of specific IEGs, may be essential to obtain new targets and strategies as treatments for fear-related disorders.

## IEG in Schizophrenia

Schizophrenia is characterized by profound cognitive deficits that are not alleviated by currently available medications. Many of these cognitive deficits involve dysfunction of the newly evolved, dorsolateral prefrontal cortex. The brains of patients with schizophrenia show atrophy in the dendrites of the pyramidal cells, particularly from dorsolateral prefrontal cortex (Weinberger et al., 1986; Bonilha et al., 2008; Konopaske et al., 2014).

It has been shown that *Egr1* expression is decreased in the dorsolateral prefrontal cortex of schizophrenic patients. This down-regulation correlates with the levels of *gad1* mRNA, which is also down-regulated in schizophrenia (Yamada et al., 2007; Pérez-Santiago et al., 2012; Kimoto et al., 2014). Antipsychotics administration, in contrast, up-regulates *Egr1* and related IEGs in frontal and striatal regions (de Bartolomeis et al., 2015a).

Searching for biological markers of schizophrenia in peripheral tissues, it was found that whole blood samples of patients suffering this disease have an increased expression of *Egr1* (Kurian et al., 2011; Cattane et al., 2015). Besides, *Egr1* and other IEGs are also associated with response to antipsychotic drugs (MacGibbon et al., 1994; Robbins et al., 2008; Bruins Slot et al., 2009; Wheeler et al., 2014; de Bartolomeis et al., 2015b; Duclot and Kabbaj, 2017b).

Changes in *c-fos* expression have been reported in schizophrenia related animal models. Acute treatment with the NMDA antagonist MK-801 produced deficits in novel object recognition, conditioned test aversion and a moderate increase in locomotion. Interestingly, animals treated with this drug showed increased levels of c-Fos expression in cortical regions associated with cognitive processing (Vishnoi et al., 2015). Subchronic, but not acute, treatment with PCP, another NMDA antagonist, produces deficits in working memory tasks and evokes a particular pattern of *c-fos* expression (Castañé et al., 2015) suggesting that there are plastic changes related to the behavioral output. Interestingly, *c-fos* expression in the mPFC does not correlate with glutamic acid decarboxylase 67 expression as does *Egr1* (Kimoto et al., 2014). CamKII is a Serine/Threonine protein that plays a key role in neural plasticity. α-CamkII heterozygous and knock out mice have working memory deficits. This behavioral deficits correlate with decrease c-Fos expression in key areas associated with working memory like mPFC and hippocampus (Matsuo et al., 2009). This changes in c-Fos expression appears to be task specific since the same α-CamkII +/– mice showed normal c-Fos expression pattern in the mPFC after fear conditioning training (Frankland et al., 2004). An interesting and new target is matrix metalloproteinase 9 (MMP-9), an enzyme activated outside the cell by proteolytic cleavage that degrades extracellular matrix. It has been implicated in synaptic plasticity and is under *c-fos* regulation (Michaluk and Kaczmarek, 2007; Michaluk et al., 2007; Huntley, 2012). Aberrant levels of MMP-9 has been observed in different psychiatric disorders, including schizophrenia and importantly, blood levels of MMP-9 or gene responsiveness to antipsychotics has been related to this disorder (Lepeta and Kaczmarek, 2015; Vafadari et al., 2016).

The interaction between Arc and dopamine appears to be bidirectional. Using a genetic model in which *arc* was deleted in the whole brain, Managò et al. (2016) found a dysregulation in the dopaminergic system with reduced dopamine levels in the cortex and increased levels in the striatum. Arc deletion induced these changes that are characteristic of a schizophrenia-related model. Consistent with this finding, the authors also found deficits in multiple domains correlated with cognitive, positive and negative associated symptoms of schizophrenia and identified a complex interaction between Arc, dopaminergic system and schizophrenia (Managò et al., 2016; Managò and Papaleo, 2017).

## Psychiatric Related Drugs and IEGs

IEGs have not only been associated directly with particular psychiatric disorders but they have also been used as markers of the specific action of different psychiatric drugs that are related. Haloperidol, a typical antipsychotic drug induces changes in *c-fos* and *Egr1* expression in the striatum and nucleus accumbens (MacGibbon et al., 1994). Olanzapine, in acute doses show increased *c-fos* expression in the medial prefrontal cortex (Robertson and Fibiger, 1996; Ohashi et al., 2000), and the locus coeruleus (Ohashi et al., 2000; Dawe et al., 2001), while a chronic treatment showed a downregulation of *Egr1* and an upregulation of FOS-like expression in the same structures (Verma et al., 2006). These results, together with other studies (Nguyen et al., 1992), suggest those antipsychotics do affect in some way IEG expression, however, they do not do it in a concerted fashion. It would be interesting to address if the IEGs’ expression pattern correlates with antipsychotics’ activity.

Antidepressants’ activity has also been analyzed in relation to their effect on IEG expression. Acute administration of fluoxetine, imipramine, LiCl, or mirtazapine produced changes in IEG expression in different regions. There were changes found for *c-fos* in the anterior insular cortex, the septum and the amygdala using different antidepressants. In the case of *Egr1* a consistent change was found only in the amygdala (Slattery et al., 2005). Chronic antidepressants can restore the up-regulation of *c-fos* in the frontal cortex following an acute stress experience (Beck and Fibiger, 1995a,b). Furthermore, chronic administration of fluoxetine and vortioxetine decreased Arc and c-Fos, but not Egr1 in the frontal cortex, hippocampus and amygdala of rodents (Waller et al., 2017).

Although these studies show changes in the expression of IEGs in apparent response to different drugs, they are correlational studies and do not address any role of these proteins in their mechanism of action. Despite this caveat, even these studies provide important information regarding circuits involved in learning and memory that can be affected in psychiatric disorders.

## Conclusion

Although the prolific use of IEGs in neuroscience, our understanding of their role in neurobiological processes remains insufficient. This is due at least to the large number of IEGs that are currently under study, but also because of the difficulty in fully comprehending their roles and their downstream effectors. Independently of this, IEGs are used as markers of synaptic activity and as that, are useful. Even more, when combined with modern techniques, like optogenetic or chemogenetics (Reijmers et al., 2007; Garner et al., 2012; Liu et al., 2012; Cowansage et al., 2014) IEGs can become powerful tools to identify activity-dependent cell populations. However, important gaps in the study of IEGs exist. As we mentioned above we still do not know much regarding the cellular effectors of these genes specifically in the Central Nervous System. Likewise, their role in memory processes requires further investigation. The lack of information regarding this particular point is surprising, especially if we consider that tools to address it have been around for decades (e.g., use of antisense or inducible genetic manipulations). A better understanding of these points becomes particularly relevant considering their role in psychiatric conditions. Understanding neuropsychiatric disorders is principally challenging since their etiology and pathophysiology are mostly unknown. Nonetheless, by focusing on the study of endophenotypes (Bearden and Freimer, 2006) together with the use of animal models (Krishnan et al., 2008; Nestler and Hyman, 2010; Anderzhanova et al., 2017), our understanding of these disorders has considerably improved in the last decade. Analyzing the role of IEGs in specific endophenotypes or animal models of psychiatric disorders could shed light on both the genetic and molecular basis of these diseases. Together with a better understanding of their biological roles, the manipulation of these genes in animal models could provide a robust model for the discovery of new pharmacological targets. In this review, we aimed to resume the literature looking at the intersection between psychiatric disorders, IEG and memory. Interestingly, we found that most of these studies were correlational; with surprisingly few studies analyzing a causal role of IEGs in psychiatric disorders. It is clear now that IEGs are much more than simple activation markers and they deserve to be analyzed as possible key participants in psychiatric conditions. Genetic manipulations that allow the temporal and spatial control of expression of these genes are powerful tools to analyze the role that IEGs have in memory and other cognitive domains in psychiatric animal models. We consider that, until now, the intersection between psychiatric models, cognition and IEG function did not receive the attention that it deserves. The manipulation of IEG expression as a means of intervention on the onset and progression of psychiatric conditions could generate new targets in the development of their treatments, targets that nowadays are completely unnoticed.

## Author Contributions

FTG, CK, JFM, JHM and NVW conceived the content, wrote and organized the manuscript.

## Conflict of Interest Statement

The authors declare that the research was conducted in the absence of any commercial or financial relationships that could be construed as a potential conflict of interest.
